# Supplementary material for: Extensive Chromatin Structure-Function Associations Revealed by Accurate 3D Compartmentalization Characterization
Source: Front Cell Dev Biol. 2022 Apr 19;10:845118. doi: 10.3389/fcell.2022.845118 (PMC9062080; doi:10.3389/fcell.2022.845118)
Supplement: Supplementary file 1 [file Table1.docx]

# Supplementary Information

# Extensive Chromatin Structure-Function Associations Revealed by Accurate 3D Compartmentalization Characterization

Zi Wen^1^^,2^, Weihan Zhang^1^, Quan Zhong^1,2^, Jinsheng Xu^1,2^, Chunhui Hou^3^, Zhaohui Steve Qin^4^ and Li Li^1,2,5^*

^1^ Hubei Key Laboratory of Agricultural Bioinformatics, College of Informatics, Huazhong Agricultural University, Wuhan, P.R. China

^2^ 3D Genomics Research Center, Huazhong Agricultural University, Wuhan, P. R. China

^3^ Department of Biology, School of Life Sciences, Southern University of Science and Technology, Shenzhen, P.R. China

^4^ Department of Biostatistics and Bioinformatics, Rollins School of Public Health, Emory University, Atlanta, USA

^5^ Hubei Hongshan Laboratory, Huazhong Agricultural University, Wuhan, P.R. China

**^*^** Correspondence: [li.li@mail.hzau.edu.cn](mailto:li.li@mail.hzau.edu.cn)


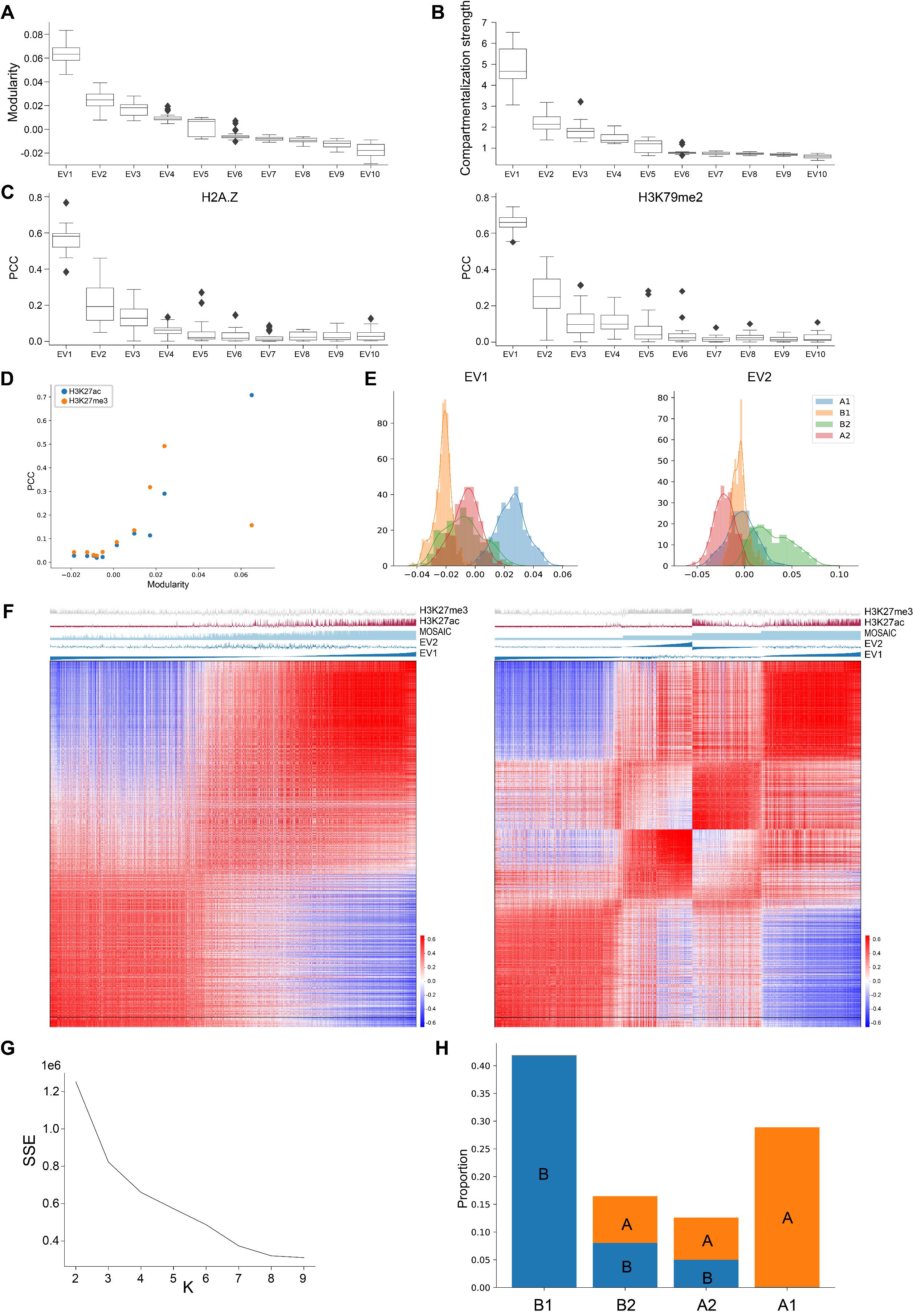


**Figure S1.** Exploration of EV1 and EV2. (**A-B**) Distribution of modularity. (**A**) and compartmentalization strength (**B**) in the top-ten EVs, sorted by modularity. (**C**) Pearson correlation coefficients between H2A.Z (left) and H3K79me2 (right) and the top-ten EVs sorted by modularity. (**D**) Scatter plot of the average Pearson correlation coefficients to histone modifications of all chromosomes versus average modularity of all chromosomes for the top-ten EVs in each chromosome, sorted by modularity. (**E**) Distribution of A1, A2, B2, and B1 on EV1 (left) and EV2 (right) in chromosome 1. (**F**) Results of the segmentation of chromosome 1 with only EV1 (left) versus the combination of combining EV1 and EV2 (right). (**G**) Sum of squared errors of results obtained by K-means clustering of EV1 and EV2 with different K. (**H**) Proportion of areas of A1, A2, B2, and B1 in the A/B compartment scenario.


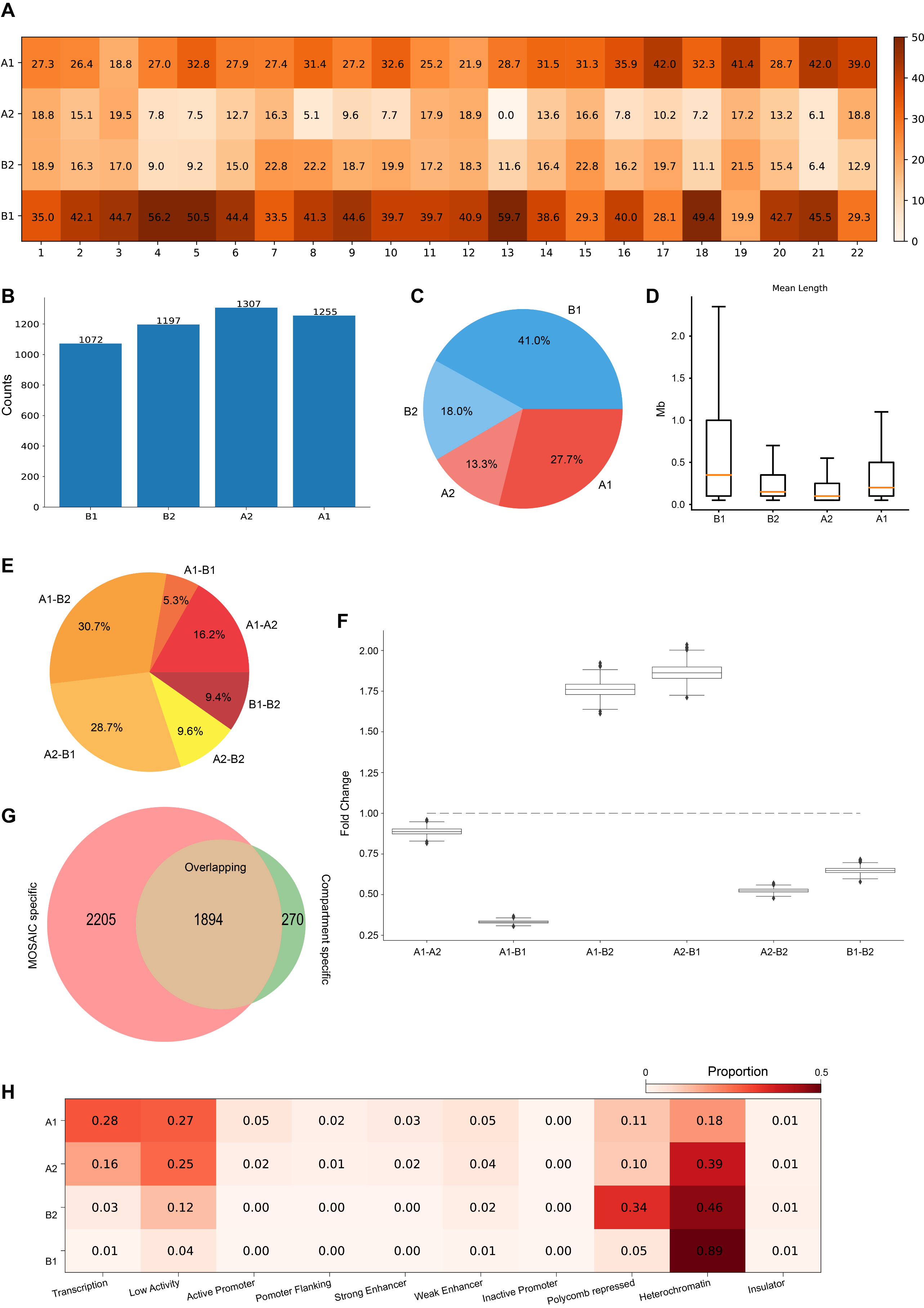


**Figure S2.** Characteristics of A2 and B2. (**A**) Compartmental state percentages for each chromosome. (**B**) The number of regions in each compartmental state. (**C**) Proportions of four compartmental states throughout the genome at 50Kb resolution. (**D**) Length distributions of four compartmental states throughout the genome at 50Kb resolution. (**E**) Proportions of border types in terms of neighboring compartmental states at 50Kb resolution. (**F**) Distribution of enrichment of true border types relative to random border types with neighborhoods shuffled 1,000 times at 50Kb resolution. (**G**) The number of borders obtained by MOSAIC and A/B compartment scenario. (**H**) Heatmap of ChromHMM annotation proportion for four compartmental states throughout the genome.


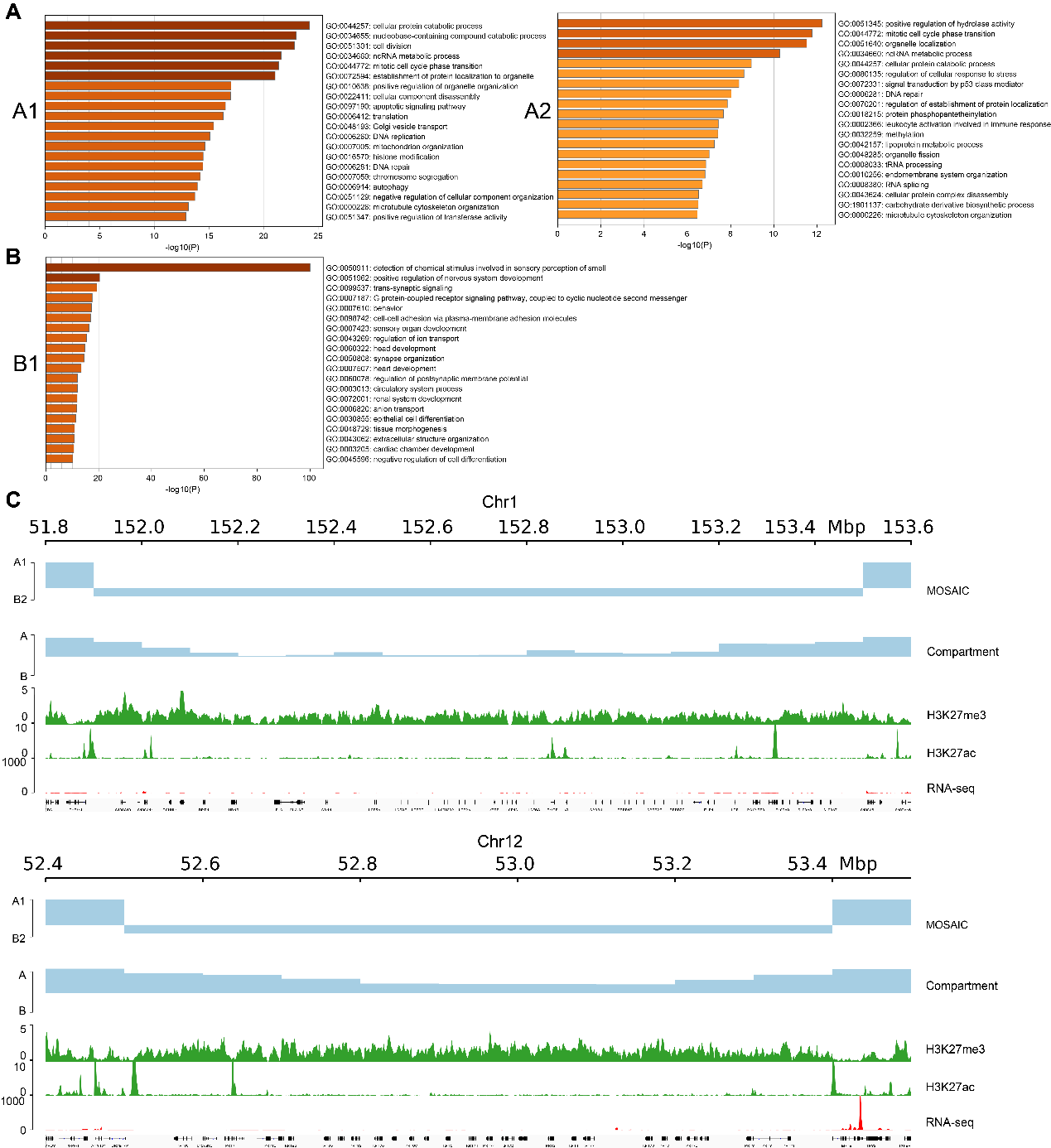


**Figure S3.** GO analysis of A1, A2, and B1. (**A**) Enriched GO terms in A1 (left) and A2 (right). (**B**) Enriched GO terms in B1. (**C**) LCE gene cluster (top) and KRT gene cluster (bottom) are all located in the B2 region identified by MOSAIC, but in the A region identified by A/B compartment scenario.


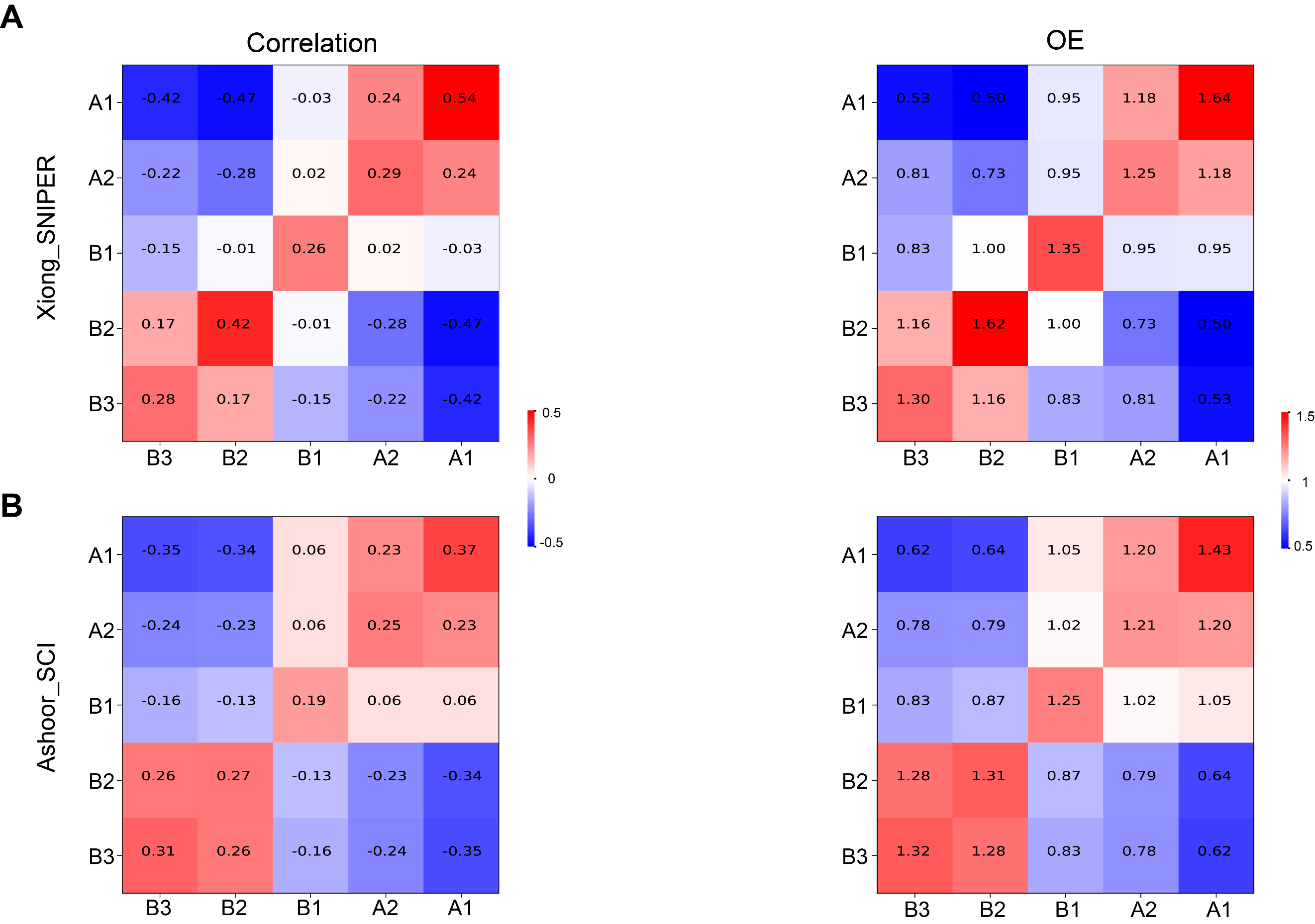


**Figure S4.** Evaluation of Xiong_SNIPER and Ashoor_SCI. (**A-B**) Heatmap of mean value of the correlation matrix (left panel) and mean value of the O/E matrix (right panel) in each compartmental state identified by Xiong_SNIPER (**A**) and Ashoor_SCI (**B**).


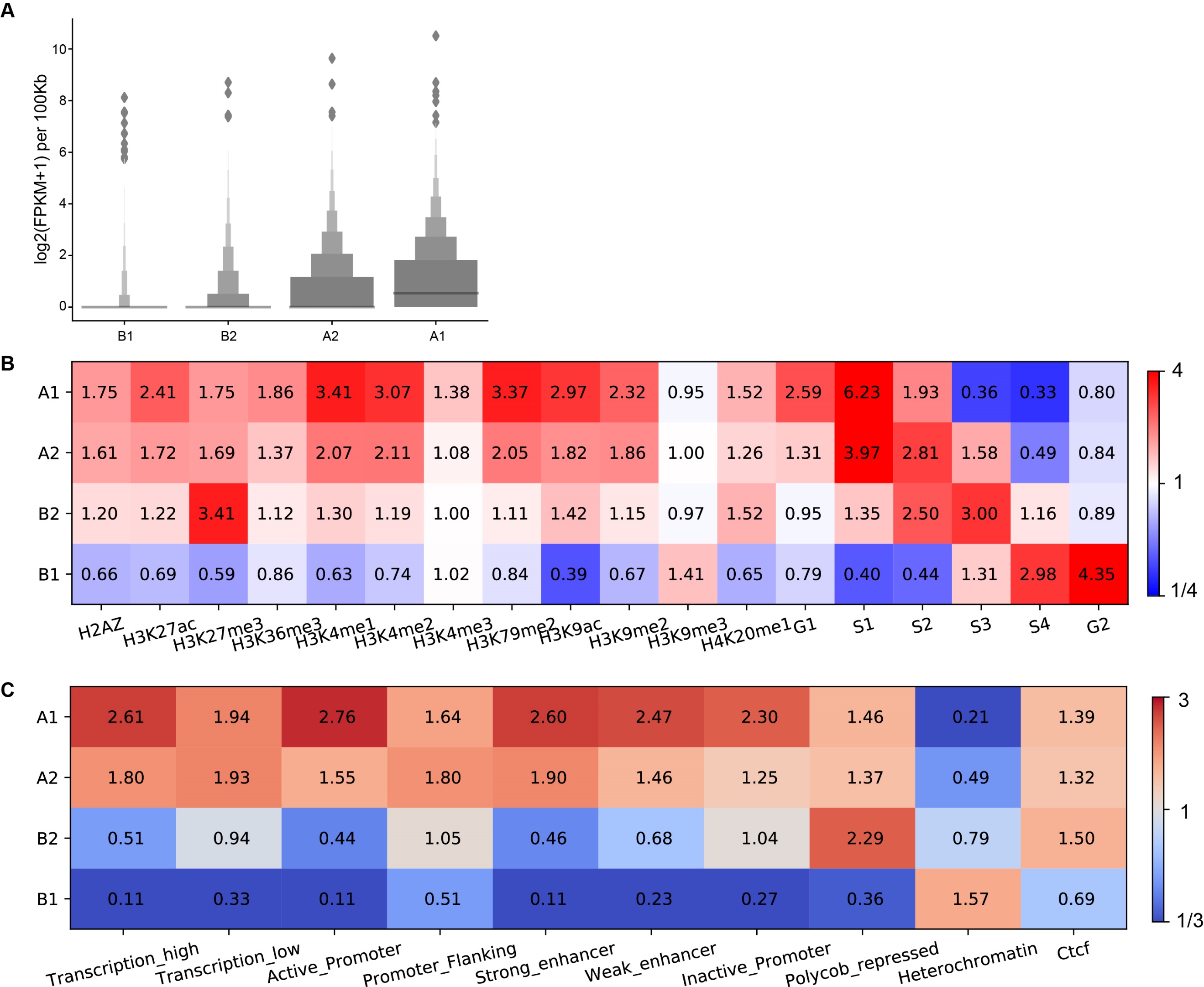


**Figure S5.** Characteristics of compartmental states identified by the MOSAIC on the K562. **(A)** Gene expression of four compartmental states throughout the genome. **(B)** Heatmap of histone mark and replication timing enrichment for four compartmental states throughout the genome. **(C)** Heatmap of ChromHMM annotation enrichment for four compartmental states throughout the genome.


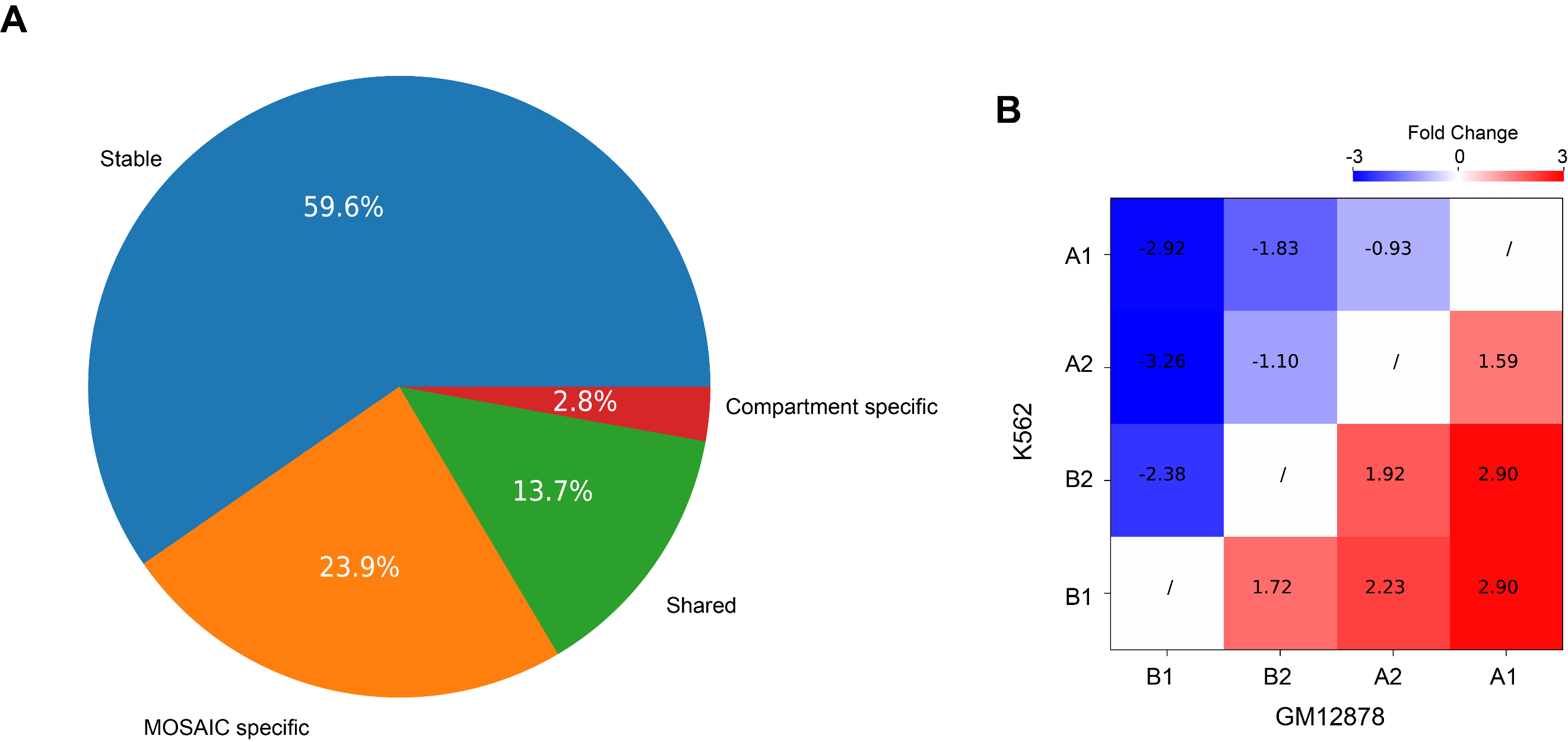


**Figure S6.** Percentage of DEGs between GM12878 and K562 in the MOSAIC scenario and the magnitude of the change. (**A**) Percentage of DEGs covered by MOSAIC and A/B compartment scenario. (**B**) The mean value of fold changes in DEGs in switches between compartmental states.
