# Supplementary material for: Extensive Chromatin Structure-Function Associations Revealed by Accurate 3D Compartmentalization Characterization
Source: Front Cell Dev Biol. 2022 Apr 19;10:845118. doi: 10.3389/fcell.2022.845118 (PMC9062080; doi:10.3389/fcell.2022.845118)
Supplement: Supplementary file 2 [file DataSheet1.docx]

# Supplementary Information

# Extensive Chromatin Structure-Function Associations Revealed by Accurate 3D Compartmentalization Characterization

Zi Wen^1^^,2^, Weihan Zhang^1^, Quan Zhong^1,2^, Jinsheng Xu^1,2^, Chunhui Hou^3^, Zhaohui Steve Qin^4^ and Li Li^1,2,5^*

^1^ Hubei Key Laboratory of Agricultural Bioinformatics, College of Informatics, Huazhong Agricultural University, Wuhan, P.R. China

^2^ 3D Genomics Research Center, Huazhong Agricultural University, Wuhan, P. R. China

^3^ Department of Biology, School of Life Sciences, Southern University of Science and Technology, Shenzhen, P.R. China

^4^ Department of Biostatistics and Bioinformatics, Rollins School of Public Health, Emory University, Atlanta, USA

^5^ Hubei Hongshan Laboratory, Huazhong Agricultural University, Wuhan, P.R. China

**^*^** Correspondence: [li.li@mail.hzau.edu.cn](mailto:li.li@mail.hzau.edu.cn)

**Data and code availability**

All downloaded data are based on the hg19 reference genome.

Compartmental conservations were obtained from 5 cell lines and 11 tissues.

The Hi-C data of 5 cell lines (GM12878, K562, IMR90, NHEK, HUVEC) were obtained from <ftp://cooler.csail.mit.edu/coolers/hg19/> in cool format but original data were from Rao et al. in GEO accession GSE63525.

The Hi-C data of 11 tissues (Adrenal, Bladder, Dorsolateral Prefrontal Cortex, Hippocampus, Lung, Ovary, Pancreas, Psoas, Right Ventricle, Small Bowel, Spleen) were obtained from GSE87112.

The signal bigWig tracks of GM12878 for histone modifications were obtained from the ENCODE consortium using the following link: <https://www.encodeproject.org/>.

The signal bigWig tracks of GM12878 and K562 for replication time data were obtained from the ENCODE consortium using the following link: <http://hgdownload.cse.ucsc.edu/goldenPath/hg19/encodeDCC/wgEncodeUwRepliSeq/>.

The RNA-seq data of GM12878, K562 in bam format were obtained from Djebali et al. in GEO accession number GSE33480.

The ChromHMM annotations of GM12878 in hg19 were obtained from ENCODE consortium using the following link: <https://genome.ucsc.edu/cgi-bin/hgFileUi?db=hg19&g=wgEncodeAwgSegmentation>.

The subcompartments identification of Rao_HMM was obtained from <https://www.ncbi.nlm.nih.gov/geo/download/?acc=GSE63525&format=file&file=GSE63525%5FGM12878%5Fsubcompartments%2Ebed%2Egz>.

The subcompartments identification of Xiong_SNIPER was obtained from <https://cmu.box.com/s/n4jh3utmitzl88264s8bzsfcjhqnhaa0>.

The subcompartments identification of Ashoor_SCI was obtained from <https://github.com/TheJacksonLaboratory/sci/tree/master/predictions>.

MOSAIC code is available on <https://github.com/WenZi0809/MOSAIC>.
